# Effect of race and ethnicity on advanced breast cancer risk prediction model performance

**DOI:** 10.1038/s41746-025-02130-y

**Published:** 2025-12-14

**Authors:** Karla Kerlikowske, Shuai Chen, Brian L. Sprague, Jeffrey A. Tice, Diana L. Miglioretti, Rebecca A. Hubbard

**Affiliations:** 1 Departments of Medicine and Epidemiology and Biostatistics, University of California, San Francisco, CA USA; 2 General Internal Medicine Section, Department of Veterans Affairs, University of California, San Francisco, CA USA; 3 Department of Public Health Sciences, University of California, Davis, CA USA; 4 Departments of Surgery and Radiology, University of Vermont, Burlington, VT USA; 5 Division of General Internal Medicine, Department of Medicine, University of California, San Francisco, CA USA; 6 Kaiser Permanente Washington Health Research Institute, Kaiser Permanente Washington, Seattle, WA USA; 7 Department of Biostatistics, Brown University, Providence, RI USA

**Keywords:** Cancer, Diseases, Oncology, Risk factors

## Abstract

Race-naive prediction modeling is proposed as a method to address algorithmic bias. We evaluated model performance by removing race and ethnicity from the Breast Cancer Surveillance Consortium 6-year cumulative advanced breast cancer risk model used for decision-making on screening frequency and supplemental imaging. Excluding race and ethnicity from the model resulted in overestimation of risk in Asian women compared to the original model [expected/observed = 1.28 (95%CI = 1.05–1.66) vs. 1.00 (95%CI = 0.82–1.29)] and underestimation of risk in Black women [expected/observed = 0.61 (95%CI = 0.53–0.70) vs. 1.00 (95%CI = 0.88–1.15)]. Advanced breast cancer among Asian women classified as intermediate/high risk increased from 6.1% to 16.7% while among Black women decreased from 75.3% to 47.5%. Fewer Black women with advanced breast cancer were identified as intermediate/high advanced breast cancer risk, with the model excluding race and ethnicity due to worse calibration. This could result in suboptimal implementation of tailored screening strategies to reduce advanced breast cancer diagnoses and breast cancer mortality.

## Introduction

There is concern that clinical risk prediction models may reinforce existing disparities or introduce new biases in the allocation of healthcare resources^1–4^. Regulatory bodies seek to understand the impact of risk prediction models that include race and ethnicity to inform the implementation of risk-targeted care, such as decision-making criteria, risk communication, and issues around decision thresholds. Race and ethnicity are often included in breast cancer risk prediction models used for prevention because breast cancer incidence and mortality vary by race and ethnicity^5–9^. Assessment of algorithmic bias, attributes intrinsic to a model that may result in differential model performance in different groups^1^, and risk model equity has primarily focused on algorithms that impact treatment, such as lung function, cardiovascular disease, and stroke, with fewer evaluating risk models for cancer prevention^10^. One study that evaluated a lung cancer risk model found removing race and ethnicity increased screening eligibility for Hispanic and Asian individuals, but decreased eligibility for Black individuals^11^, while a prostate cancer risk model found adding a race coefficient provides a more accurate prediction of prostate cancer risk among Black men^12^.

Advanced breast cancer is a surrogate for breast cancer mortality^13^, making advanced breast cancer risk a clinically relevant measure to assess when discussing screening strategies with women. The Breast Cancer Surveillance Consortium (BCSC) developed the first actionable advanced breast cancer risk model to guide decisions on screening interval and the need for supplemental imaging in individuals with high advanced breast cancer risk (https://tools.bcsc-scc.ucdavis.edu/AdvBC6yearRisk/#/)^5^. The model includes race and ethnicity, and demonstrated that advanced breast cancer rates are twofold higher in Black compared to White women. Overall, the model has shown that in a screening population, most women (69%) are at low or average advanced breast cancer risk with annual or biennial screening, suggesting these women can undergo biennial screening to minimize the likelihood of screening harms associated with annual screening. Twelve percent of women are at intermediate advanced breast cancer risk with biennial screening and average risk with annual screening, suggesting they might benefit from receiving annual screening, and 17% are at intermediate or high advanced cancer risk with annual or biennial screening, suggesting consideration of supplemental imaging^5^. Given the 2024 USPSTF recommendation for biennial screening mammography for all women aged 40–74 years^14^, and the federal breast density law enacted in September 2024 requiring radiology practices to inform the 47% of women with dense breasts that they should consider of supplemental imaging^15^, the actionable advanced breast cancer risk calculator is uniquely positioned to guide clinical decisions on screening interval and supplemental imaging.

To assess the impact of including race and ethnicity in the BCSC advanced breast cancer risk model on algorithmic bias and model equity, we evaluated the effect of removing race and ethnicity from the model. We hypothesize that removing race and ethnicity from the advanced breast cancer risk model will reduce calibration and discrimination across race and ethnicity groups.

## Results

The study cohort included 931,186 women aged 40–74 years undergoing 2,542,382 annual and 752,049 biennial screening mammograms who developed 1110 and 760 advanced breast cancers and 7297 and 4237 non-advanced breast cancers, respectively. Compared with women screened biennially, women screened annually tended to be older, have a family history of breast, and history of breast biopsy (Table 1).

**Table 1:** Characteristics of women undergoing annual and biennial screening^5^

Characteristics	Annual (*n* = 2,542,382)					Biennial (*n* = 752,049)				
	No advanced breast cancer^a^		Advanced breast cancer^b^			No advanced breast cancer^a^		Advanced breast cancer^b^		
	No.	Column %	No.	Column %	Row %	No.	Column %	No.	Column %	Row %
Screening examinations^c^	2,541,267	99.96	1110	—^d^	0.04	751,285	99.90	760	—^d^	0.10
Age, years										
40–49	633,753	24.9	198	17.8	0.03	206,148	27.4	159	20.9	0.08
50–59	895,381	35.2	396	35.7	0.04	280,198	37.3	272	35.8	0.10
60–69	759,869	29.9	389	35.0	0.05	206,723	27.5	251	33.0	0.12
70–74	252,264	9.9	127	11.4	0.05	58,216	7.7	78	10.3	0.13
Race and ethnicity										
Asian, non-Hispanic	237,823	9.4	72	6.5	0.03	102,763	13.7	88	11.6	0.09
Black, non-Hispanic	228,253	9.0	192	17.3	0.08	66,261	8.8	103	13.6	0.16
Hispanic	116,719	4.6	48	4.3	0.04	47,418	6.3	40	5.3	0.08
White, non-Hispanic	1,797,793	70.7	736	66.3	0.04	487,396	64.9	493	64.9	0.10
Other/Multiple race, non-Hispanic	42,626	1.7	22	2.0	0.05	18,856	2.5	19	2.5	0.10
Unknown	118,053	4.6	40	3.6	0.03	28,591	3.8	17	2.2	0.06
Menopausal										
No	632,688	24.9	237	21.4	0.04	203,065	27.0	184	24.2	0.09
Yes	1,454,112	57.2	708	63.8	0.05	416,189	55.4	463	60.9	0.11
Unknown^e^	454,467	17.9	165	14.9	0.04	132,031	17.6	113	14.9	0.09
1st degree family history of breast cancer^f^										
No	2,000,401	78.7	802	72.3	0.04	632,629	84.2	614	80.8	0.10
Yes	443,781	17.5	270	24.3	0.06	96,810	12.9	127	16.7	0.13
Unknown	97,085	3.8	38	3.4	0.04	21,846	2.9	19	2.5	0.09
History of breast biopsy										
None (no prior biopsy)	1,948,017	76.7	717	64.6	0.04	627,322	83.5	565	74.3	0.09
Prior biopsy, benign diagnosis unknown	388,553	15.3	262	23.6	0.07	90,950	12.1	153	20.1	0.17
Non-proliferative	142,949	5.6	91	8.2	0.06	24,025	3.2	30	3.9	0.12
Proliferative without atypia	52,226	2.1	31	2.8	0.06	7974	1.1	10	1.3	0.13
Proliferative with atypia	9522	0.4	9	0.8	0.09	1014	0.1	2	0.3	0.20
BI-RADS breast density										
Almost entirely fat	236,336	9.3	40	3.6	0.02	71,988	9.6	25	3.3	0.03
Scattered fibroglandular densities	1,069,779	42.1	360	32.4	0.03	296,890	39.5	235	30.9	0.08
Heterogeneously dense	981,763	38.6	512	46.1	0.05	292,213	38.9	312	41.1	0.11
Extremely dense	207,731	8.2	109	9.8	0.05	62,774	8.4	74	9.7	0.12
Unknown	45,658	1.8	89	8.0	0.19	27,420	3.6	114	15.0	0.41
Body mass index, kg/m^2^										
Underweight (<18.5)	25,654	1.0	10	0.9	0.04	8190	1.1	5	0.7	0.06
Normal (18.5–24.9)	700,038	27.5	231	20.8	0.03	216,024	28.8	177	23.3	0.08
Overweight (25.0–29.9)	480,818	18.9	190	17.1	0.04	150,198	20.0	173	22.8	0.12
Obese I (30.0–34.9)	252,175	9.9	102	9.2	0.04	81,359	10.8	103	13.6	0.13
Obese II/III (≥35.0)	182,208	7.2	103	9.3	0.06	65,515	8.7	53	7.0	0.08
Unknown	900,374	35.4	474	42.7	0.05	229,999	30.6	249	32.8	0.11

^a^Includes non-advanced breast cancers. American Joint Committee on Cancer (AJCC), Breast Imaging Reporting and Data System (BI-RADS).

^b^Invasive cancer AJCC 8th edition prognostic pathologic stage II or higher within 12 or 24 months of screening mammography.

^c^Subsequent screening examinations.

^d^Not applicable.

^e^Unknown includes perimenopausal, surgical menopause, periods stopped for other (not natural or surgical) reason, and other unknown due to not available information and were imputed to be either premenopausal or postmenopausal.

^f^Defined as first-degree relative (mother, sister, or daughter) with breast cancer.

### Calibration

In annual screeners, excluding race and ethnicity from the model resulted in overestimation of advanced breast cancer risk in Asian women compared to the original model [expected/observed = 1.28 (95%CI = 1.05–1.66) vs. 1.00 (95%CI = 0.82–1.29)] and an underestimation of advanced breast cancer risk in Black women [expected/observed=0.61 (95%CI = 0.53–0.70) vs. 1.00 (95%CI = 0.88–1.15)] and Other/Multiple race women [expected/observed=0.82 (95%CI = 0.70–0.99) vs. 1.00 (95%CI = 0.85–1.21)] (Table 2; Fig. S1). Biennial screeners had similar results except excluding race and ethnicity from the model resulted in overestimation of advanced breast cancer risk in Hispanic women compared to the original model [expected/observed=1.14 (95%CI = 0.92–1.52) vs. 1.00 (95%CI = 0.80–1.33)] while the model excluding race and ethnicity and the original model were both well calibrated for Other/Multiple race and White women undergoing biennial screening (Table 1; Fig. S2).

**Table 2:** Calibration for annual and biennial screeners, including and excluding race and ethnicity from the advanced breast cancer risk model

Race and ethnicity	Expected 1-yr (E) (%)		Observed 1-yr (O) (%)	Expect/observed (E/O) annual screens (95% CI)		Expected 2-yr (E) (%)		Observed 2-yr (O) (%)	Expect/observed (E/O) biennial screens (95% CI)	
	Model including race and ethnicity	Model excluding race and ethnicity		Model including race and ethnicity	Model excluding race and ethnicity	Model including race and ethnicity	Model excluding race and ethnicity		Model including race and ethnicity	Model excluding race and ethnicity
Asian, non-Hispanic	0.030	0.039	0.030	1.00 (0.82, 1.29)	1.28 (1.05, 1.66)	0.085	0.093	0.085	1.00 (0.80, 1.32)	1.10 (0.88, 1.45)
Black, non-Hispanic	0.083	0.051	0.083	1.00 (0.88, 1.15)	0.61 (0.53, 0.70)	0.151	0.112	0.151	1.00 (0.84, 1.22)	0.74 (0.63, 0.91)
Hispanic	0.041	0.040	0.041	1.00 (0.84, 1.24)	0.97 (0.82, 1.20)	0.083	0.096	0.083	1.00 (0.80, 1.33)	1.14 (0.92, 1.52)
Other/Multiple race, non-Hispanic	0.052	0.043	0.052	1.00 (0.85, 1.21)	0.82 (0.70, 0.99)	0.101	0.100	0.101	1.00 (0.82, 1.29)	0.99 (0.81, 1.28)
White, non-Hispanic	0.040	0.044	0.040	1.00 (0.84, 1.24)	1.08 (0.91, 1.34)	0.099	0.102	0.099	1.00 (0.82, 1.29)	1.02 (0.84, 1.32)

### Discrimination

Overall discrimination was slightly higher for the model including race and ethnicity than the model excluding race and ethnicity [AUC = 0.682 (95%CI = 0.670–0.694) vs. 0.677 (95%CI = 0.665–0.689)] (Table 3). Discrimination was similar across racial and ethnic groups for the two models, with discrimination slightly higher for Asian, Black and Other/Multiple race women in the model excluding vs. including race and ethnicity and slightly lower for Hispanic and White women.

**Table 3:** Area under the receiver operating characteristic curve for the advanced breast cancer risk model with and without race and ethnicity

	Model including race and ethnicity (95% CI)	Model excluding race and ethnicity (95% CI)
Overall	0.682 (0.670–694)	0.677 (0.665–689)
Race and ethnicity		
Asian, non-Hispanic	0.658 (0.614–703)	0.664 (0.620–709)
Black, non-Hispanic	0.653 (0.620–685)	0.656 (0.623–689)
Hispanic	0.666 (0.608–725)	0.663 (0.604–722)
Other/Multiple race, non-Hispanic	0.679 (0.612–746)	0.695 (0.624–767)
White, non-Hispanic	0.684 (0.669–698)	0.682 (0.668–697)

### Absolute risk

Based on predicted advanced breast cancer risk under annual screening, the percentage of Asian women estimated at intermediate/high risk when excluding race and ethnicity from the advanced breast cancer risk model increased from 3.4% to 10.2% (Table 4). By contrast, the percentage of Black women estimated at intermediate/high advanced breast cancer risk when excluding race and ethnicity from the advanced breast cancer risk model decreased from 58.5% to 24.1% as did the percentage of Other/Multiple race women 27.9% to 17.0% (Table 4). White and Hispanic women had similar percentages of intermediate/high advanced breast cancer risk, whether including or excluding race and ethnicity from the advanced breast cancer risk model.

**Table 4:** Prevalence of high, intermediate, average, low and very low cumulative risk of advanced breast cancer after 6 years of annual screening in models including and excluding race and ethnicity

		Percentage of women by race and ethnicity					
Risk Category	Overall risk % (mean)	Overall percentage of women	Asian,non-Hispanic	Blac, non-Hispanick	Hispanic	Other/Multiple race, non-Hispanic	White, non-Hispanic
High (>0.658), including race and ethnicity^a^	0.865	4.1	0.1	20.2	2.7	4.7	1.8
High (>0.623) excluding race and ethnicity^b^	0.792	3.3	1.0	5.0	2.2	3.0	3.4
Difference	–0.073	–0.8	+0.9	−15.2	−0.5	−1.7	+1.6
Intermediate (0.380–0.658) including race and ethnicity^a^	0.480	15.5	3.3	38.3	13.6	23.2	12.7
Intermediate (0.376–0.623) excluding race and ethnicity^b^	0.467	15.0	9.2	19.1	11.6	14.0	15.5
Difference	–0.013	–0.05	+5.9	−19.2	−2.0	−9.2	+2.8
Average (0.172–0.379) including race and ethnicity^a^	0.255	49.0	45.7	34.1	43.9	50.4	53.1
Average (0.175–0.376) excluding race and ethnicity^b^	0.260	50.7	57.9	50.4	49.2	50.3	50.3
Difference	+0.005	+1.7	+12.2	+16.3	+5.3	–0.1	−2.8
Low (0.090–0.171), including race and ethnicity^a^	0.132	24.5	41.2	6.6	28.1	18.8	25.6
Low (0.091–0.175), excluding race and ethnicity^b^	0.136	24.3	26.6	20.3	28.1	25.3	24.0
Difference	+0.004	–0.2	−14.6	+13.7	0	+6.5	−1.6
Very low (<0.089), including race and ethnicity^a^	0.068	6.9	9.7	0.9	11.7	2.9	6.8
Very low (<0.091) excluding race and ethnicity^b^	0.071	6.8	5.3	5.2	9.0	7.4	6.7
Difference	+0.003	–0.1	−4.4	+4.3	−2.7	+4.5	–0.1

^a^Risk threshold based on distribution of risk in combined sample of annual and biennial screeners from the original model, including race and ethnicity; High risk; >95th percentile, intermediate risk; >75th and ≤95th percentile, average risk; >25th and ≤75th percentile, low risk; >5th and ≤25th percentile, very low risk; ≤5th percentile. Risk and prevalence adjusted by US population weights and standardized to the same population for annual and biennial^5^.

^b^Risk threshold based on distribution of risk in combined sample of annual and biennial screeners from the model excluding race and ethnicity; High risk; >95th percentile, intermediate risk; >75th and ≤95th percentile, average risk; >25th and ≤75th percentile, low risk; >5th and ≤25th percentile, very low risk; ≤5th percentile. Risk and prevalence adjusted by US population weights and standardized to the same population for annual and biennial^5^.

**Fig. 1:**
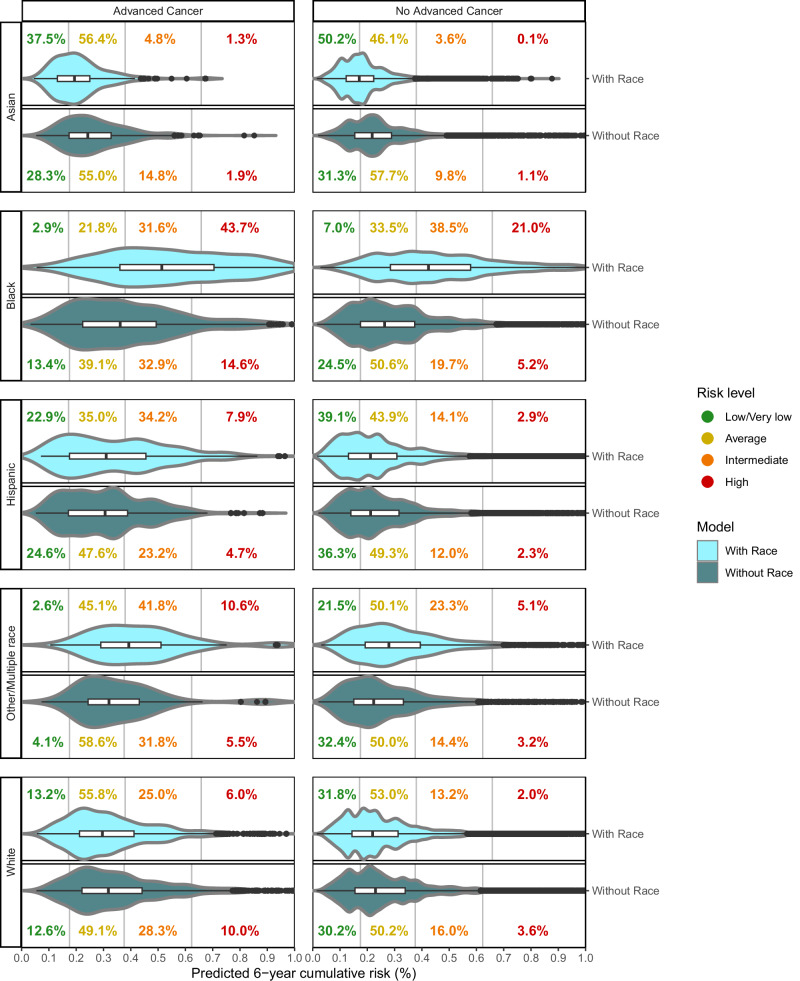
Violin plots showing the distribution of predicted cumulative risk of advanced breast cancer after 6 years of annual screening using models that include and exclude race and ethnicity, with prevalence of high, intermediate, average, low and very low cumulative risk, stratified by races and women diagnosed and not diagnosed with advanced breast cancer. Risk threshold based on distribution of risk in the combined sample of annual and biennial screeners of all races for each model: High risk; >95th percentile; intermediate risk,>75th and ≤95th percentile; average risk; >25th and ≤75th percentile; low risk; >5th and ≤25th percentile; very low risk; ≤5th percentile. Risk and prevalence adjusted by US population weights and standardized to the same population for annual and biennial.

Among women diagnosed with advanced breast cancer undergoing annual screening, a higher percentage of Black, Hispanic and Other/Multiple race women were identified as intermediate/high advanced breast cancer risk with the model including vs. excluding race and ethnicity (Fig. 1). By contrast, a higher percentage of Asian and White women are identified as intermediate/high advanced breast cancer risk with the model excluding vs. including race and ethnicity. For example, the percentage of advanced breast cancer classified as intermediate/high risk among Black women decreased from 75.3% to 47.5% and in Asian women increased from 6.1% to 16.7%. Black, Hispanic, and Other/Multiple race women diagnosed with advanced breast cancer have a lower estimated median absolute advanced breast cancer risk with the advanced breast cancer risk model excluding race and ethnicity vs. a model including race and ethnicity, while Asian and White women have higher estimated advanced breast cancer risk (Fig. 1, Table 5).

**Table 5:** Median (IQR) values for cumulative 6-year predicted advanced breast cancer risk using models including or excluding race, stratified by race and ethnicity and advanced breast cancer status

		Median (IQR) of cumulative 6-year predicted risk (%)			
		Annual		Biennial	
Race/ethnicity	Model	Advanced breast cancer	No advanced breast cancer	Advanced breast cancer	No advanced breast cancer
Asian, non-Hispanic	Including race and ethnicity	0.194 (0.131, 0.250)	0.171 (0.124, 0.223)	0.303 (0.190, 0.410)	0.224 (0.159, 0.315)
	Excluding race and ethnicity	0.245 (0.174, 0.331)	0.218 (0.154, 0.289)	0.307 (0.244, 0.447)	0.264 (0.191, 0.341)
Black, non-Hispanic	Including race and ethnicity	0.577 (0.384, 0.848)	0.438 (0.296, 0.608)	0.520 (0.374, 0.744)	0.420 (0.255, 0.560)
	Excluding race and ethnicity	0.366 (0.230, 0.515)	0.265 (0.179, 0.376)	0.382 (0.286, 0.532)	0.308 (0.203, 0.422)
Hispanic	Including race and ethnicity	0.322 (0.175, 0.458)	0.210 (0.130, 0.309)	0.327 (0.214, 0.472)	0.241 (0.159, 0.330)
	Excluding race and ethnicity	0.313 (0.190, 0.435)	0.213 (0.141, 0.317)	0.367 (0.242, 0.510)	0.266 (0.176, 0.380)
Other/Multiple race, non-Hispanic	Including race and ethnicity	0.393 (0.290, 0.511)	0.279 (0.191, 0.395)	0.379 (0.283, 0.517)	0.291 (0.185, 0.399)
	Excluding race and ethnicity	0.320 (0.243, 0.431)	0.223 (0.151, 0.331)	0.389 (0.284, 0.486)	0.286 (0.187, 0.399)
White, non-Hispanic	Including race and ethnicity	0.298 (0.213, 0.417)	0.220 (0.144, 0.313)	0.361 (0.268, 0.488)	0.284 (0.189, 0.385)
	Excluding race and ethnicity	0.323 (0.224, 0.461)	0.230 (0.155, 0.341)	0.373 (0.267, 0.503)	0.289 (0.190, 0.399)

Among women not diagnosed with advanced breast cancer undergoing annual screening, a higher percentage of Asian and White women were incorrectly categorized as intermediate/high advanced breast cancer risk for the model excluding vs. including race and ethnicity. A lower percentage of Black, Hispanic and Other/Multiple race women not diagnosed with advanced breast cancer were categorized as intermediate/high advanced breast cancer risk when race and ethnicity were excluded from the model. Black and Other/Multiple race women who did not develop advanced breast cancer have a lower estimated median absolute advanced breast cancer risk with the advanced breast cancer risk model excluding race and ethnicity, while Asian and White women have a higher estimated advanced breast cancer risk (Fig. 1, Table 5).

Biennial screeners have similar results to annual screeners, except Hispanic and other/multiple race women have a slightly greater percentage of women identified as intermediate/high risk of advanced breast cancer with the model excluding race and ethnicity (Table 5, Table S1, and Fig. S3).

## Discussion

This study examined whether removing race and ethnicity from the BCSC advanced breast cancer risk model, used for decision-making on screening frequency and supplemental imaging, impacted the accurate categorization of women as intermediate/high advanced breast cancer risk. Calibration was optimal for all racial and ethnic groups when race and ethnicity was included in the advanced breast cancer risk model, whereas excluding race and ethnicity from the model resulted in overestimation of risk in some racial and ethnic groups and underestimation in others. We found that including race and ethnicity in the model categorized a higher proportion of Black, Hispanic and Other/Multiple race women who would develop advanced breast cancer as intermediate/high advanced breast cancer risk compared to a model excluding race and ethnicity. By contrast, including race and ethnicity in the advanced breast cancer risk model categorized a lower proportion of Asian and White women who develop advanced breast cancer as intermediate/high advanced breast cancer risk compared to the model excluding race and ethnicity. Given Black, Hispanic and Other/Multiple race women have a higher proportion of advanced breast cancer than Asian women, a model including race and ethnicity will identify more of these women who may benefit from annual screening and/or supplemental imaging.

Black women have the largest proportion of women diagnosed with advanced breast cancer who would not be identified as having intermediate/high advanced breast cancer risk if race and ethnicity are removed from the advanced breast cancer risk model. Including race and ethnicity in the model would identify the largest number of Black women diagnosed with advanced breast cancer as having intermediate/high advanced breast cancer risk for consideration of annual screening and/or supplemental imaging; however, it would also identify the largest number of Black women not diagnosed with advanced breast cancer who might be similarly considered for annual screening and/or supplemental imaging. By contrast, Asian women have the lowest rate of advanced breast cancer, so a small number of women diagnosed with advanced breast cancer would not be identified as intermediate/high advanced breast cancer risk if race and ethnicity are included in the advanced breast cancer risk model. Excluding race and ethnicity from the model would identify more Asian women who will develop advanced breast cancer, who could consider annual screening and/or supplemental imaging, as well as more women who will not develop advanced breast cancer, but who could be recommended for annual screening and/or supplemental imaging. Thus, there is a trade-off between appropriately offering more frequent screening and/or supplemental imaging to women with the highest rate of developing advanced breast cancer and inappropriately offering more frequent screening and/or supplemental imaging to women who will not develop advanced breast cancer, leading to false-positive tests and benign biopsies^16^. By including race and ethnicity in the advanced breast cancer risk model, a greater number of Black, Hispanic and Other/Multiple race women at intermediate/high advanced breast cancer risk who will develop advanced breast cancer could be offered annual screening and/or supplemental imaging at the detriment of a smaller number of Asian women being identified as intermediate/high advanced breast cancer risk who will develop advanced breast cancer.

A study of colon cancer patients examined whether including race and ethnicity as a predictor in a colorectal cancer recurrence risk algorithm results in racial and ethnic differences in model accuracy that could potentially lead to unequal treatment^17^. The authors found calibration was worse when excluding race and ethnicity, and this could lead to inappropriate care recommendations for some racial and ethnic groups. A study of a colon cancer risk prediction model found that adjusting for race and ethnicity compensated for data quality differences for key predictors and increased the fraction of Black participants among the predicted high-risk group eligible for screening^18^. Another study evaluating a lung cancer risk model found that removing race and ethnicity increased screening eligibility for Hispanic and Asian individuals but decreased eligibility for Black individuals^11^. A cross-sectional study found removing race and ethnicity from the Breast Cancer Risk Assessment Tool resulted in substantial miscalibration, overestimating invasive cancer risk in Asian women^19^. As with our advanced breast cancer risk model, excluding vs. including race and ethnicity from the model resulted in reduced calibration and had differential effects by race and ethnicity, with the magnitude of the effect greatest for Black women.

We studied a large, diverse, population-based sample of women undergoing annual or biennial screening, which makes our results most applicable to regular screeners. Some estimated confidence intervals are wide due to small sample sizes, resulting in imprecise risk estimates. Our study does not address optimal advanced breast cancer risk thresholds or individual preferences for advanced breast cancer risk thresholds. Harms were not assessed, but published studies have reported the cumulative risk of false-positive mammography and biopsy by screening interval and reported almost 2-fold greater harms with annual vs. biennial screening and supplemental imaging^16,20,21^. We were unable to separate Pacific Islander women from other racial and ethnic groups, which is an important modification for future model versions, given that Pacific Islander women are at increased risk of advanced breast cancer^22^.

Determining advanced breast cancer risk in women with risk factors for advanced breast cancer (Black race, obesity, and heterogeneously or extremely dense breasts) and offering annual screening and/or supplemental imaging to those at intermediate/high advanced breast cancer risk could lead to a decrease in advanced breast cancer diagnoses and breast cancer mortality. Supplemental imaging and more frequent screening among those at elevated breast cancer risk have been associated with reductions in advanced cancer rates and breast cancer mortality^23,24^. Mutation carriers who undergo supplemental MRI screening have been shown to have reduced advanced breast cancer rates compared to those who undergo mammography alone^23^. Modeling studies have shown that more deaths are averted from breast cancer among women at high breast cancer risk screened annually vs. biennially^24^. Recently, supplemental ultrasound among women with heterogeneously or extremely dense breasts has been shown to have higher cancer detection rates for women with high advanced breast cancer risk as opposed to high invasive cancer risk^25^. Taken together, these studies suggest that assessing advanced breast cancer risk and tailoring screening frequency and supplemental imaging use could reduce the risk of an advanced breast cancer. However, studies are needed and underway to directly evaluate the association of advanced breast cancer risk assessment and clinical management strategies with the development of advanced breast cancer and breast cancer mortality.

We found removing race and ethnicity from the advanced breast cancer risk model worsened calibration for Black and Asian women and had a small impact on discrimination. Removing race and ethnicity from the model resulted in identifying fewer Black, Hispanic and Other/Multiple race women who would develop advanced breast cancer as having intermediate/high advanced breast cancer risk—women who may have benefited from annual screening and/or supplemental imaging. Including race and ethnicity in the advanced breast cancer risk model identifies a high proportion of women who develop advanced breast cancer as having high advanced breast cancer risk, supporting retaining race and ethnicity in the model. Thus, given our model is well calibrated, which is a necessary criterion for model fairness^26^, risk models with modest discriminatory accuracy (AUCs of 0.6–0.7) such as ours still provide a useful perspective for individual counseling and for weighing the harms and benefits of preventive clinical interventions^27^. For example, a 59-year-old Black postmenopausal obese woman with heterogeneously dense breasts undergoing annual or biennial mammography would be at high risk of advanced breast cancer and could consider supplemental imaging. Whereas the same woman who was of normal weight with scattered fibroglandular density would be at average risk of advanced cancer with annual or biennial screening.

Incorporating new risk factors associated with advanced breast cancer risk, such as mammographic calcifications^28^, social vulnerability index^29^, and/or artificial intelligence-derived measures^30^ into the advanced breast cancer risk model with the goal of improving discriminatory accuracy will require re-evaluation of whether model calibration is maintained for all women.

## Methods

### Study setting and data sources

Data were from the BCSC mammography registries (http://www.bcsc-research.org/index.html), whose demographics are comparable to the US population^31–33^. We prospectively collected data on women’s characteristics and mammography from radiology facilities. Breast cancer diagnoses and tumor characteristics were obtained by linking women to pathology databases; regional Surveillance, Epidemiology, and End Results programs; and state tumor registries. Deaths were obtained by linking to state death records. Registries and the Statistical Coordinating Center (SCC) received Institutional Review Board approval for active or passive consenting processes or a waiver of consent to enroll participants, link data, and perform analyses. All procedures were Health Insurance Portability and Accountability Act compliant, and registries and the SCC received a Federal Certificate of Confidentiality and other protections for the identities of women, physicians, and facilities.

### Participants

We used the data used to create the BCSC advanced breast cancer risk model as previously described^5^. In brief, screening mammograms were defined based on radiologist’s reported clinical indication. To reflect women routinely screened, we identified 3,507,682 screening mammograms performed from January 2005 through December 2017 among women aged 40–74 years with a mammogram 11–30 months earlier. Annual screening was defined as having a prior mammogram within 11–18 (mean = 13.8) months, and biennial screening was defined as having a prior mammogram within 19–30 (mean = 23.7) months. We excluded screens from women with either a breast cancer history, mastectomy or lobular carcinoma in situ, screening mammograms that were either unilateral, preceded by mammography within 9 months, performed with a screening ultrasound on the same day, or screening MRI occurred 12 months before or after leaving 3,294,431 annual or biennial screens. The model is available on the web (https://tools.bcsc-scc.ucdavis.edu/AdvBC6yearRisk/#/) and can be downloaded via the iPhone or Google Play app stores. In the last two years, the web page has been visited over 4200 times, the free iOS App has been downloaded >2000 times, and the free Android App has been downloaded >300 times.

### Measures, definitions, outcomes

We collected demographic and breast health history from self-administered surveys at screening and/or extracted from electronic health records. Women self-reported race and ethnicity separately which we classify into the following categories with Hispanic as a separate group and all race groups are non-Hispanic: Hispanic and non-Hispanic African American/Black, Asian, White, Other/Multiple race (non-Hispanic Native American/Alaskan Native, Native Hawaiian or Pacific Islander), or with two or more reported races, or other). Radiologists categorized breast density during clinical interpretation using Breast Imaging Reporting and Data System (BI-RADS)^34^ density categories: almost entirely fat, scattered fibroglandular densities, heterogeneously dense, and extremely dense. Postmenopausal women were those with both ovaries removed, whose periods had stopped naturally, who were current postmenopausal hormone therapy users, or who were age 60 or older. Premenopausal women reported a period within the last 180 days or birth control hormone use. Perimenopausal women were not sure if their periods had stopped, or their last menstrual period was 180-364 days prior^35–37^. Body mass index (BMI) was categorized as <18.5 kg/m^2^ = underweight, 18.5–24.9 kg/m^2^ = normal weight, 25.0–29.9 kg/m^2^ = overweight, 30.0–34.9 kg/m^2^ = obese I, and ≥35.0 kg/m^2^ = obese II/III^38^.

We grouped prior benign diagnoses abstracted from clinical pathology reports based on the highest grade as proliferative with atypia > proliferative without atypia > non-proliferative using published taxonomy^39–41^ or as unknown if a woman reported a prior biopsy with no available BCSC pathology result.

Mammograms were linked to invasive breast cancer or ductal carcinoma in situ diagnoses within 12 months after annual and 24 months after biennial mammography. We calculated the American Joint Committee on Cancer, 8th edition prognostic, pathologic stage^42^ using anatomic staging elements, tumor grade, and estrogen, progesterone, and human epidermal growth factor receptor status as in the original BCSC advanced breast cancer risk model^5^.

### Statistical approach

The screening mammogram was the unit of analysis unless otherwise specified. We characterized mammograms associated with no advanced breast cancer or advanced breast cancer for annual and biennial screens according to risk factors. We estimated absolute advanced breast cancer risk and competing events (death or early-stage cancer) using logistic regression. For the original BCSC advanced breast cancer model^5^, separate models were fit by menopausal status (premenopausal versus postmenopausal) and screening interval and included age (linear and quadratic), race and ethnicity, first-degree breast cancer family history, history of benign biopsy, BMI, and breast density. To evaluate the impact of excluding race and ethnicity, we further fitted similar models but excluded race and ethnicity and compared the performance of the two models. Before model fitting, 15 imputed values for each missing variable were generated using MICE (multiple imputation chained equations)^43^. For each covariate combination, risk scores from a single screening round were estimated by averaging over the 15 risk scores estimated from each imputed dataset. Full details of MICE methods used in developing the model have been previously published^5^. Model calibration, which tests if predicted risks are numerically accurate, was estimated by the ratio of expected to observed number of advanced breast cancers after one screening round overall and for race/ethnic groups. Model discriminatory accuracy, which tests if the model can tell who is at high versus low risk, was summarized overall and for racial/ethnic groups using the area under the receiver operating characteristic curve (AUC) estimated with 5-fold cross-validation to compare predicted to observed risk after one screening round (Fig. S4).

Cumulative 6-year advanced breast cancer risks after six annual and three biennial screens were estimated using a discrete time survival model based on the fitted logistic regression models for each screening round while considering competing risks of death or early-stage cancer^44^. Advanced breast cancer could occur after any annual or biennial screen during the six-year follow-up period. Mean six-year cumulative risks and interquartile ranges for annual and biennial screening were standardized to the U.S. population of women based on age, race and ethnicity, and breast cancer family history by weighting the overall study cohort^45,46^. According to the six-year cumulative advanced breast cancer risk, women were categorized into five risk levels for each model: high, >95th percentile; intermediate, >75th to ≤95th percentile; average, >25th and ≤75th percentile; low, >5th and ≤25th percentile; very low, ≤5th percentile. The percentages of women in each risk level were compared between the two models for the overall cohort and by race/ethnic groups. Violin plots including boxes representing the middle 50% of cumulative advanced breast cancer risk were used to compare the two models, stratified by race/ethnic groups and advanced breast cancer status.

Data were analyzed using R version 4.3.3 (R Foundation for Statistical Computing, Vienna, Austria) and SAS version 9.4 (SAS Institute, Cary, NC).

## Supplementary information

Supplementary information

## Data Availability

The de-identified dataset underlying this manuscript will be shared upon email request to the BCSC SCC ([kpwa.scc@kp.org]). Some variables in the dataset may require approval from state agencies to allow third-party data sharing.
