# Supplementary material for: Effect of race and ethnicity on advanced breast cancer risk prediction model performance
Source: NPJ Digit Med. 2025 Dec 14;8:771. doi: 10.1038/s41746-025-02130-y (PMC12717068; doi:10.1038/s41746-025-02130-y)

## Supplementary Materials

**Table S1**. Prevalence of high, intermediate, average, low and very low cumulative risk of advanced cancer after 6 years of biennial screening for models that include and exclude race/ethnicity.

| Risk Category | Overall risk % (mean) | Overall percentage of women | Percentage of women by race and ethnicity: Asian | Black | Hispanic | Other/ Multiple race | White |
|---|---|---|---|---|---|---|---|
| High (>0.658) including race and ethnicity[a] | 0.846 | 5.9 | 1.5 | 19.8 | 2.2 | 5.3 | 4.5 |
| High (>0.623) excluding race and ethnicity[b] | 0.781 | 6.7 | 3.0 | 8.8 | 5.2 | 6.3 | 7.0 |
| Difference | -0.065 | +0.8 | +1.5 | -11.0 | +3.0 | +1.0 | +2.5 |
| Intermediate (0.380-0.658) including race and ethnicity[a] | 0.485 | 24.5 | 14.0 | 40.3 | 17.2 | 24.7 | 24.0 |
| Intermediate (0.376-0.623) excluding race and ethnicity[b] | 0.466 | 25.0 | 18.2 | 30.8 | 22.6 | 24.3 | 25.1 |
| Difference | -0.019 | +0.05 | +4.2 | -9.5 | +5.4 | -0.4 | +1.1 |
| Average (0.172-0.379) including race and ethnicity[a] | 0.269 | 50.6 | 54.6 | 32.4 | 53.5 | 48.2 | 53.1 |
| Average (0.175-0.376) excluding race and ethnicity[b] | 0.269 | 49.2 | 61.8 | 43.6 | 49.1 | 48.2 | 49.1 |
| Difference | +0.000 | -1.4 | +7.2 | +11.2 | -4.4 | 0.0 | -4.0 |
| Low (0.090-0.171) including race and ethnicity[a] | 0.136 | 15.9 | 25.8 | 6.3 | 21.7 | 18.1 | 15.5 |
| Low (0.091-0.175) excluding race and ethnicity[b] | 0.139 | 15.8 | 14.5 | 13.9 | 18.8 | 17.6 | 15.7 |
| Difference | +0.003 | -0.1 | -11.3 | +7.6 | -2.9 | -0.5 | +0.2 |
| Very low (<0.089) including race and ethnicity[a] | 0.069 | 3.1 | 4.1 | 1.3 | 5.4 | 3.7 | 2.9 |
| Very low (<0.091) excluding race and ethnicity[b] | 0.070 | 3.3 | 2.5 | 2.8 | 4.2 | 3.6 | 3.2 |
| Difference | +0.001 | +0.2 | -1.6 | +1.5 | -1.2 | -0.1 | +0.3 |

[a]Risk threshold based on distribution of risk in combined sample of annual and biennial screeners from the original model including race/ethnicity; High-risk; >95$^{th}$ percentile, intermediate risk; >75$^{th}$ and ≤95$^{th}$ percentile, average risk; >25$^{th}$ and ≤75$^{th}$ percentile, low risk; >5$^{th}$ and ≤25$^{th}$ percentile, very low risk; ≤5$^{th}$ percentile. Risk and prevalence adjusted by US population weights and standardized to same population for annual and biennial.
[b]Risk threshold based on distribution of risk in combined sample of annual and biennial screeners from the model excluding race/ethnicity; High-risk; >95$^{th}$ percentile, intermediate risk; >75$^{th}$ and ≤95$^{th}$ percentile, average risk; >25$^{th}$ and ≤75$^{th}$ percentile, low risk; >5$^{th}$ and ≤25$^{th}$ percentile, very low risk; ≤5$^{th}$ percentile. Risk and prevalence adjusted by US population weights and standardized to same population for annual and biennial.

**Figure S1.** Calibration results for advanced cancer risk model for annual screening by race and ethnicity for model including and excluding race and ethnicity with graph showing 95% confidence intervals of the observed and expected rate in each risk decile group.

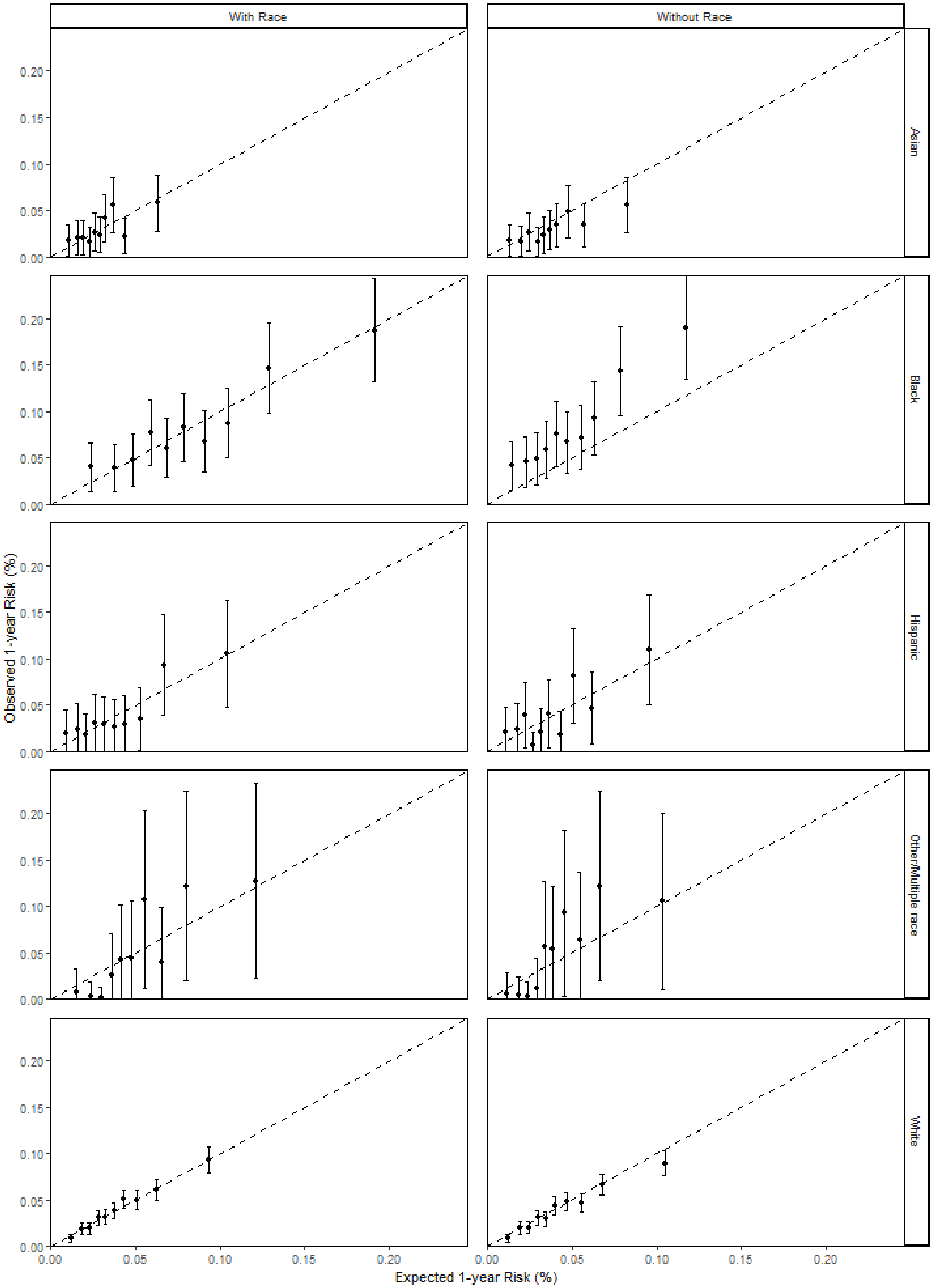

**Figure S2.** Calibration results for advanced cancer risk model for biennial screening by race and ethnicity for model including and excluding race and ethnicity with graph showing 95% confidence intervals of the observed and expected rate in each risk decile group.

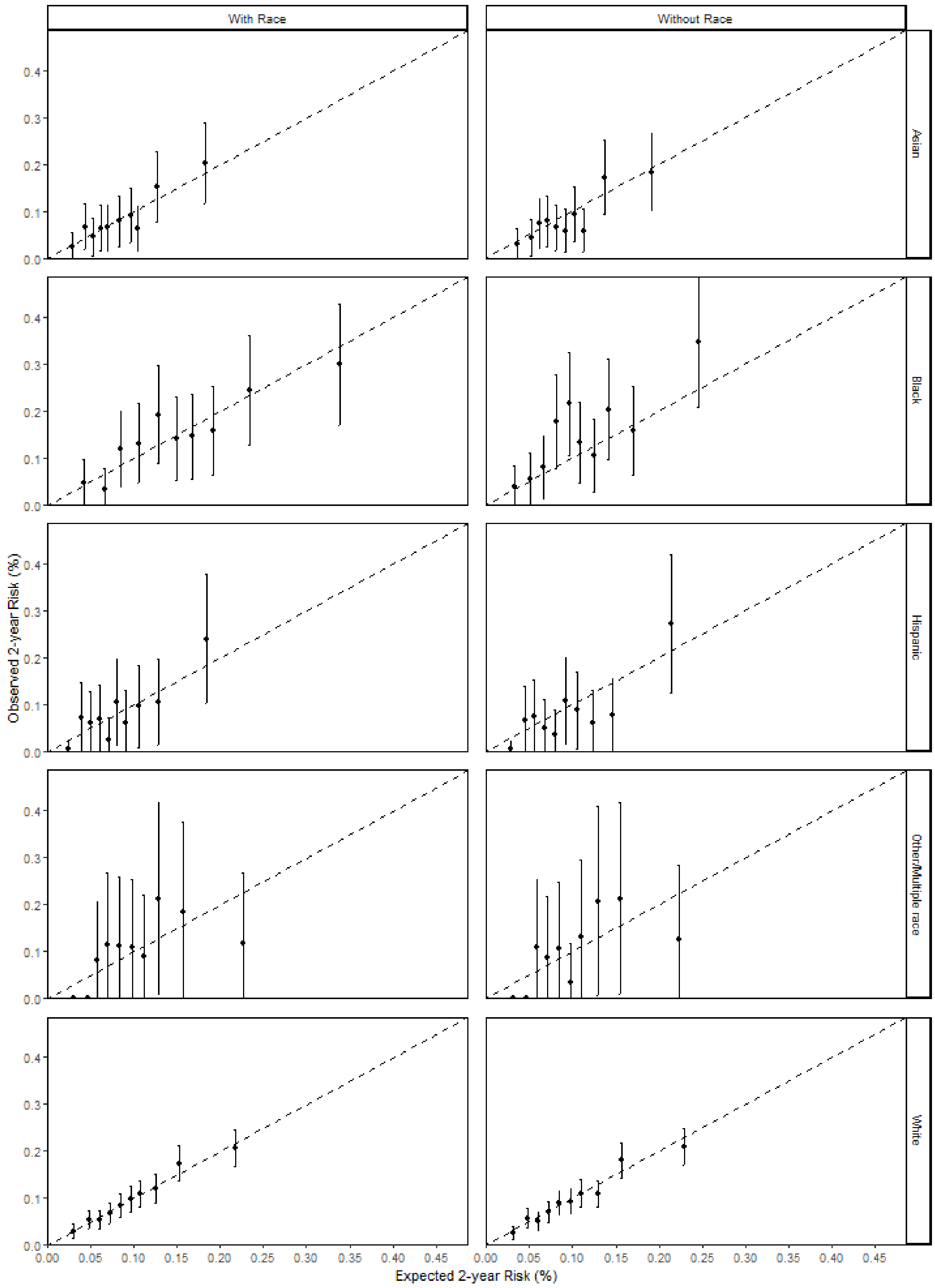

**Figure S3.** Violin plots showing the distribution of predicted cumulative risk of advanced cancer after 6 years of biennial screening using models that include and exclude race and ethnicity, with prevalence of high, intermediate, average, low and very low cumulative risk, stratified by races and women diagnosed and not diagnosed with advanced breast cancer. Risk threshold based on distribution of risk in combined sample of annual and biennial screeners of all races for each model; High-risk; >95th percentile, intermediate risk; >75th and ≤95th percentile, average risk; >25th and ≤75th percentile, low risk; >5th and ≤25th percentile, very low risk; ≤5th percentile. Risk and prevalence adjusted by US population weights and standardized to same population for annual and biennial.

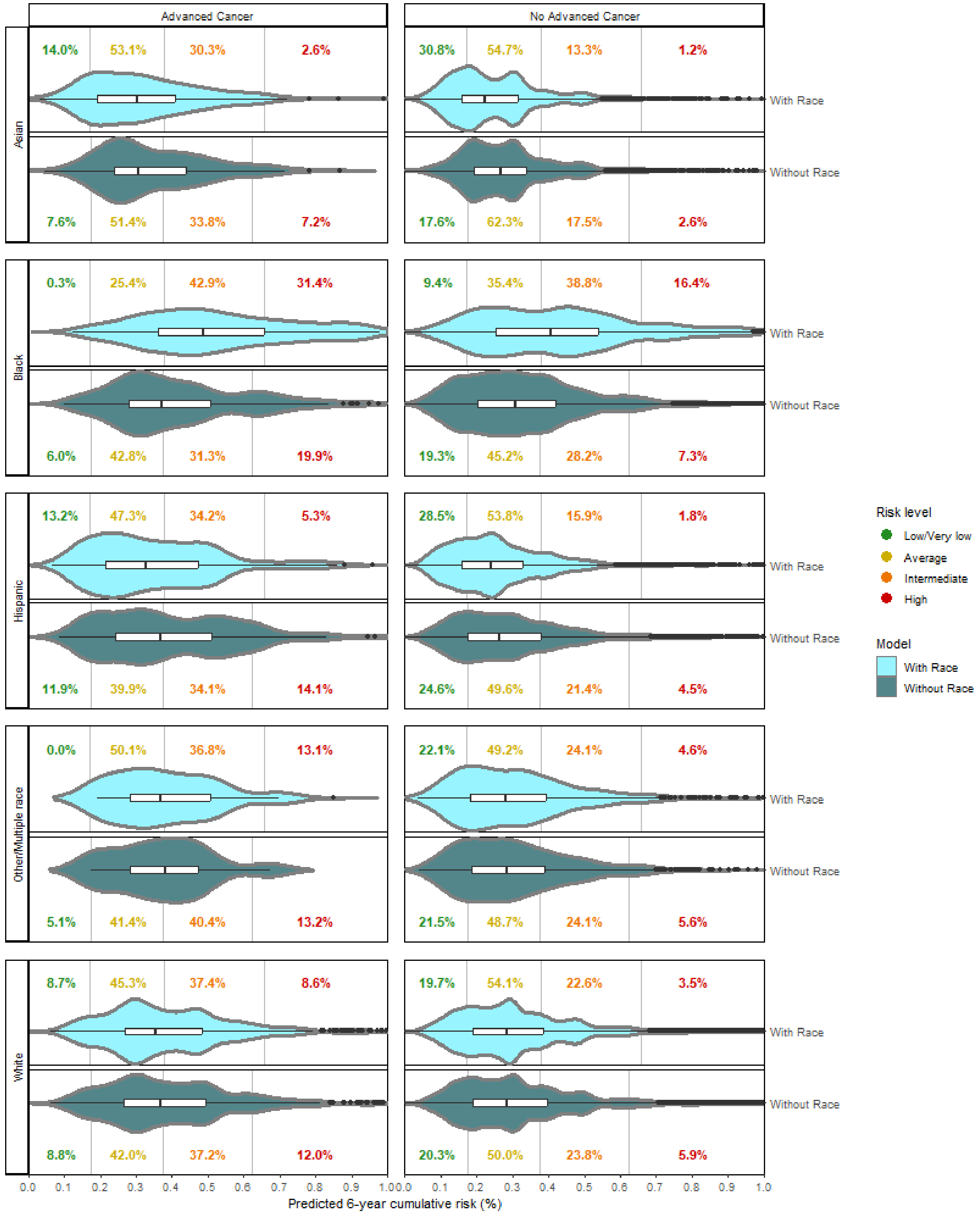

**Figure S4.** Flow-diagram delineating study methods

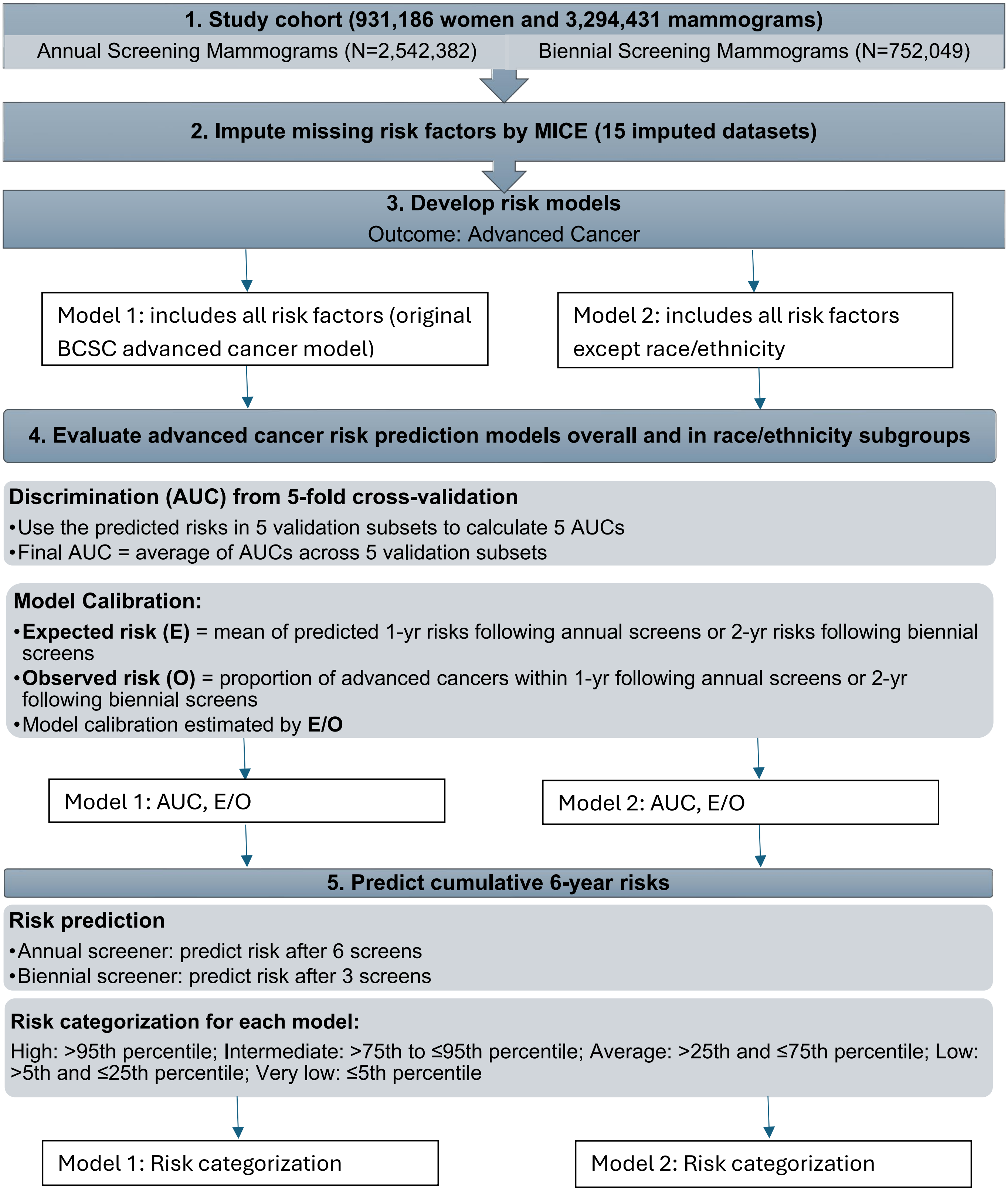

Supplement: Supplementary file 1 — Supplementary information [file 41746_2025_2130_MOESM1_ESM.pdf]
